# Large exonic deletions in *POLR3B* gene cause POLR3-related leukodystrophy

**DOI:** 10.1186/s13023-015-0279-9

**Published:** 2015-06-05

**Authors:** Mariana Gutierrez, Isabelle Thiffault, Kether Guerrero, Gabriel Á. Martos-Moreno, Luan T. Tran, William Benko, Marjo S. van der Knaap, Rosalina M. L. van Spaendonk, Nicole I. Wolf, Geneviève Bernard

**Affiliations:** Departments of Pediatrics, Neurology and Neurosurgery, Montreal Children’s Hospital, McGill University Health Center, 1001 Boulevard Décarie, Montréal, QC H4A 3J1 Canada; Center for Pediatric Genomic Medicine, Children’s Mercy Hospitals, 2420 Pershing Rd, suite 421, Kansas City, MO 64108 USA; Department of Pediatrics & Pediatric Endocrinology, Hospital Infantil Universitario Niño Jesús, Instituto de Investigación La Princesa, Madrid, Spain; Department of Pediatrics, Universidad Autónoma de Madrid, CIBER Fisiopatología Obesidad y Nutrición, Instituto de Salud Carlos III, Madrid, Spain; WellSpan Pediatric Neurology, WellSpan Medical Group, York, PA USA; Department of Child Neurology, VU University Medical Center, and Neuroscience Campus Amsterdam, Amsterdam, The Netherlands; Department of Clinical Genetics, VU University Medical Center, Amsterdam, The Netherlands

**Keywords:** 4H leukodystrophy, Pol III (POLR3)-related leukodystrophy, *POLR3A*, *POLR3B*, Deletion

## Abstract

POLR3-related (or 4H) leukodystrophy is an autosomal recessive disorder caused by mutations in *POLR3A* or *POLR3B* and is characterized by neurological and non-neurological features. In a small proportion of patients, no mutation in either gene or only one mutation is found. Analysis of the *POLR3B* cDNA revealed a large deletion of exons 21–22 in one case and of exons 26–27 in another case. These are the first reports of long deletions causing POLR3-related leukodystrophy, suggesting that deletions and duplications in *POLR3A* or *POLR3B* should be investigated in patients with a compatible phenotype, especially if one pathogenic variant has been identified.

## Findings

POLR3-related leukodystrophy or 4H (Hypomyelination, Hypodontia, Hypogonadotropic Hypogonadism) leukodystrophy (MIM#607694) is a hypomyelinating leukodystrophy with typical onset in early childhood [1–3]. Neurological features include motor delay or regression, cerebellar and pyramidal features, and, later in the course of the disease, cognitive regression. Non-neurological features include a variety of dental and hormonal abnormalities, and myopia [1, 3]. Brain MRI shows hypomyelination, i.e. variable (hypo-, hyper- or iso- intense) signal on T1-weighted images and hyperintense signal of the white matter on T2-weighted images compared to grey matter structures [4], with relative preservation of myelination (i.e. T2-hypointensity), of specific structures, with or without cerebellar atrophy and thinning of the corpus callosum [3, 5, 6]. 4H is caused by recessive mutations in *POLR3A* (MIM#614258) [7–10] or *POLR3B* (MIM#614366), encoding respectively the largest and second largest subunits of the DNA-directed RNA polymerase III (POLR3). POLR3 is responsible for the transcription of transfer RNAs and other small RNAs essential for cellular processes [11]. Amino acid changes in the protein domains of POLR3A or POLR3B suggest a direct interference with DNA binding, a modification of the catalytic cleft structure, or a change in protein interactions of POLR3 subunits [9, 10]. Current knowledge suggests that mutations are uncovered in the vast majority of 4H cases. A small percentage of cases remain negative or have only one mutation after sequencing analysis [3, 8]. To date, no large deletion or duplication has been reported in 4H cases [3, 9].

We here report the first large exonic deletions in patients with clinical and radiological diagnosis of 4H. The first index patient is a 15-year-old girl, first evaluated by a child neurologist at the age of 19 months for unstable stance and delayed walking. She was born from healthy non-consanguineous parents after a normal pregnancy and delivery. At initial evaluation, the patient had normal height, weight and head circumference. Neurological examination was significant for mild hypotonia as well as truncal ataxia. Patient evolution and annual examinations revealed delayed decidual teeth eruption, slowly progressive gait ataxia and halted pubertal development at Tanner Stage IV with primary amenorrhea. At the time, LHRH stimulation showed lack of LH/FSH pulsatility. The second patient, a boy, was born at term by elective caesarean section after a pregnancy sustained by progesterone in the context of having had three prior miscarriages of unknown cause. Congenital hip dislocation was first treated conservatively, and then corrected surgically. In the second year of life, delay of gross and fine motor skills became obvious. He developed frank ataxia and a mild pyramidal syndrome. Eruption of upper medial incisors was delayed. He developed myopia and short stature. Neurological deterioration led to death at age 8 years. MRI of both patients were compatible with 4H leukodystrophy.

**Fig. 1:**
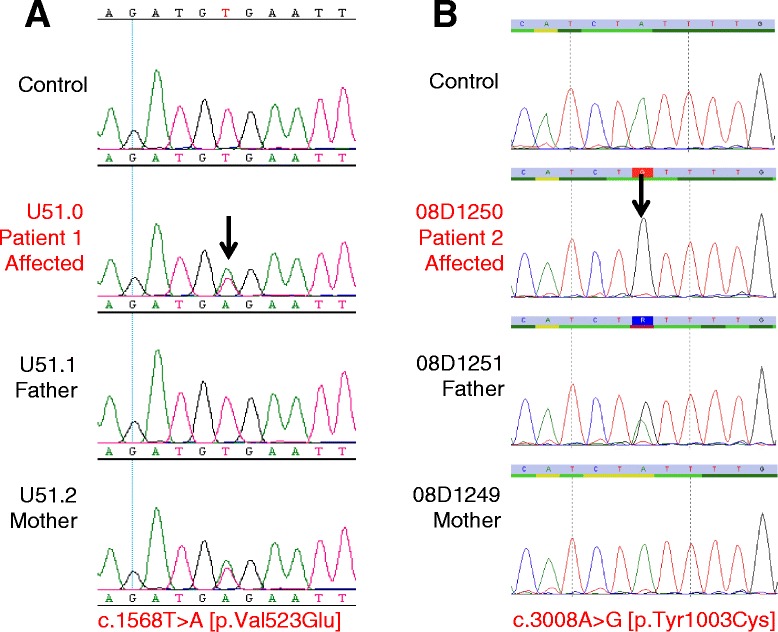
Sanger sequencing of *POLR3B* genomic coding sequence and segregation analysis in families. **a** Patient 1 was heterozygous for a maternally-inherited pathogenic variant, c.1568 T > A (p.Val523Glu), located in exon 15 of *POLR3B* gene. Father was negative for the variant. The Val523Glu variant has not been reported in the general population databases. While not validated for clinical use, multiple *in silico* analyses predict that this variant is disease-causing. **b** Patient 2 was found apparently homozygous for a paternally-inherited pathogenic variant, c.3008G > A (p.Tyr1003Cys), located in exon 26 of *POLR3B* gene. Father was a heterozygous carrier but mother was negative for the variant, suggesting a deletion on the other allele. The Tyr1003Cys variant has not been reported in the general population databases. While not validated for clinical use, multiple *in silico* analyses predict that this variant is disease-causing

**Fig. 2:**
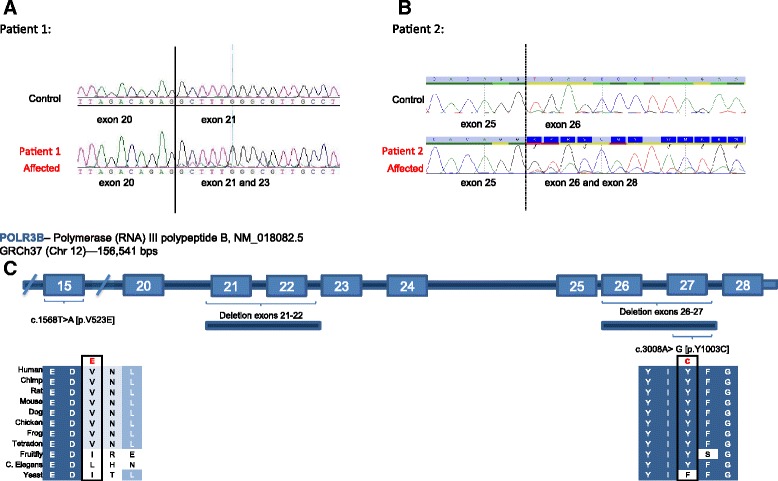
cDNA amplification and sequencing of fragments encompassing the putative *POLR3B* exonic deletions. **a** Patient 1: RNA isolation was performed using standard method and followed by reverse transcription-polymerase chain reaction (RT-PCR). Amplification of the fragment of complement DNA (cDNA) encompassing exons 20 to 23 revealed the presence of a putative deletion. The sequence in the overlapping reading frame were showing a normal cDNA PCR product of 630 bp, covering exons 20 to 23. The second sequence of a reduced product of 353 bp was encompassing exons 20 to 23, suggesting the complete or partial deletion of exons 21 and 22. **b** Patient 2: RNA isolation was performed using standard method and followed by reverse transcription-polymerase chain reaction (RT-PCR). Amplification of the fragment of complement DNA (cDNA) encompassing the exon 25 to 28 revealed the presence of a putative deletion. The sequence in the overlapping reading frame were showing a normal cDNA PCR product of 634 bp, covering exons 25 to 28. The second sequence of a reduced product of 346 bp was encompassing exons 25 and 28, suggesting the complete or partial deletion of exons 26 and 27. **c** Scheme depicts the large deletions in *POLR3B* gene characterized in our index patients with classical clinical and radiological findings of 4H

Both patients underwent sequencing of all exons and intron-exon boundaries of *POLR3A* and *POLR3B*. Patient 1 was heterozygous for a maternally-inherited variant, c.1568 T > A (p.Val523Glu), located in exon 15 of *POLR3B* (Figs. 1a and 2c). The Val523Glu have been reported several times in the literature as pathogenic [1]. Patient 2 was apparently homozygous for a missense variant, c.3008A > G (p.Tyr1003Cys), located in exon 26 of *POLR3B* (Figs. 1b and 2c). Parental testing failed to uncover the Tyr1003Cys in the mother suggesting the presence of a large deletion on the other allele or uniparental isodisomy. No mutation in *POLR3A* was uncovered in either patient.

**Fig. 3:**
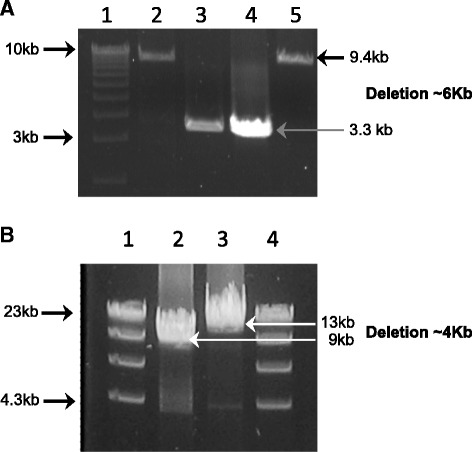
Long-Range PCR of the putative *POLR3B* exonic deletions and sequencing of the breakpoints. **a.** Patient 1: Long-Range (LR- PCR) was performed on genomic DNA using exon-specific forward and reverse primers for exons 20 and 23 respectively. The expected fragment size of this region was estimated at 10 kb (Control DNA; Lane 2 and U51.2; Lane 5), however the proband LR-PCR amplification generated a smaller DNA fragment with average product length of approximately 3 kb, as indicated by the arrow (U51.0; Lane 3). This 3 kb DNA fragment was also seen in the LR-PCR amplification of paternal DNA (U51.1; Lane 4), but not of maternal DNA (U51.2; Lane 5). Lane 1 is the molecular size marker. Sanger sequencing of the abnormal fragment identified in both the proband and the unaffected father allowed mapping of the breakpoint on the 5’ at position 106, 751, 658 on chromosome 12, and the breakpoint at the 3’ was at position 106, 857, 267 (data not shown). No homology was found at either of the deletion breakpoints. Tandem Repeats finder, QuadParser and Repeat Masker databases were used for sequence motif analysis. These tools failed to identify motif, suggesting that homologous recombination events were unlikely. The exonic deletions of *POLR3B* appear to have arisen by the simple rejoining of non-homologous DNA ends during double stranded break repair. **b.** Patient 2: Long-Range (LR- PCR) was performed on genomic DNA using intron-specific forward and reverse primers for introns 25 and 27 respectively. The expected fragment size of this region was estimated at 13 kb (Control DNA; Lane 3), however the proband LR-PCR amplification generated a smaller DNA fragment with average product length of approximately 9 kb, as indicated by the arrow (08D1250; Lane 2). Lane 1 and 4 is the molecular size marker

Further studies were conducted in order to uncover exonic deletion in our index patients. In patient 1, RNA analysis revealed the presence of a heterozygous deletion encompassing exons 21 and 22 (Fig. 2a and c). Subsequently, long-range genomic PCR using exon-specific primers for exons 20 and 23, confirmed a ~6Kb paternally-inherited deletion in patient 1 (Figs. 2c and 3a). In patient 2, RNA analysis revealed the presence of a heterozygous deletion encompassing exons 26 and 27 (Fig. 2b and c). Subsequently, long-range genomic PCR using exon-specific primers for exons 25 and 27 respectively, confirmed a ~4Kb maternally-inherited deletion. Since no homology was found at the deletion breakpoints, the *POLR3B* exonic deletions appear to have arisen by the simple rejoining of non-homologous DNA ends during double stranded break repair.

The clinical picture in these patients is typical of 4H. The presence of a deletion together with a missense mutation reiterates the idea that complete loss of *POLR3B* is lethal [1]. Patients with compound heterozygous mutations have shown no significant differences in the clinical course of deterioration as compared to homozygous affected patients, except for the patients homozygous for the common *POLR3B* mutation (c.1568 T > A, p.Val523Glu), whose clinical symptoms are significantly milder [3, 10]. This could be, at least partly, attributed to the debilitating effect of the missense mutation on the formation of multiplex complexes [9]. In summary, our findings highlight that multi-modal approaches in mutation screening of *POLR3A* and *POLR3B* genes may be required for clinically suspected 4H cases when Sanger sequencing of coding elements is negative or reveals only one mutation.

### Ethics and consent statement

The project was approved by the research ethics committee of the Montreal Children Hospital (11–105-PED) and the institutional review board of the VU University Medical Center, Neuroscience Campus Amsterdam, Netherlands. Written informed consent was obtained from the patients’ legal guardians.

## Abbreviations

4H: Hypomyelination Hypodontia Hypogonadotropic Hypogonadism
DNA: Deoxyribonucleic Acid
FSH: Follicle stimulating hormone
LH: Luteinizing hormone
LHRH: Luteinizing hormone-releasing hormone
LR-PCR: Long-Range Polymerase Chain Reaction
MRI: Magnetic Resonance Imaging
PCR: Polymerase Chain Reaction
POLR3: DNA-directed RNA polymerase III
POLR3A: DNA-Directed RNA Polymerase III Subunit A
POLR3B: DNA-Directed RNA Polymerase III Subunit B
RNA: Ribonucleic Acid

## References

[1] Bernard G, Vanderver A (1997). Pol III-related leukodystrophies (august 2012) in: GeneReviews at GeneTests: medical genetics information resource [database online].

[2] Potic A, Brais B, Choquet K, Schiffmann R, Bernard G (2012). 4H syndrome with late-onset growth hormone deficiency caused by *POLR3A* mutations. Arch Neurol.

[3] Wolf NI, Vanderver A, van Spaendonk RML, Schiffmann R, Brais B, Bugiani M (2014). Clinical spectrum of 4H leukodystrophy caused by *POLR3A* and *POLR3B* mutations. Neurology.

[4] Schiffmann R, van der Knaap MS (2009). Invited article: an MRI-based approach to the diagnosis of white matter disorders. Neurology.

[5] Steenweg ME, Vanderver A, Blaser S, Bizzi A, de Koning TJ, Mancini GM, van der Knaap MS (2010). Magnetic resonance imaging pattern recognition in hypomyelinating disorders. Brain.

[6] La Piana R, Tonduti D, Dressman HG, Schmidt JL, Murnick J, Brais B, Vanderver A (2014). Brain magnetic resonance imaging (MRI) pattern recognition in Pol III-related leukodystrophies. J Child Neurol.

[7] Saitsu H, Osaka H, Sasaki M, Takanashi JI, Hamada K, Yamashita A, Matsumoto N (2011). Mutations in *POLR3A* and *POLR3B* encoding RNA polymerase III subunits cause an autosomal-recessive hypomyelinating leukoencephalopathy. Am J Hum Genet.

[8] Bernard G, Chouery E, Putorti ML, Tétreault M, Takanohashi A, Carosso G, Brais B (2011). Mutations of *POLR3A* encoding a catalytic subunit of RNA polymerase Pol III cause a recessive hypomyelinating leukodystrophy. Am J Hum Genet.

[9] Tétreault M, Choquet K, Orcesi S, Tonduti D, Balottin U, Teichmann M, Bernard G (2011). Recessive mutations in POLR3B, encoding the second largest subunit of Pol III. Cause a rare hypomyelinating leukodystrophy. Am J Hum Genet.

[10] Daoud H, Tétreault M, Gibson W, Guerrero K, Cohen A, Gburek-Augustat J, Bernard G (2013). Mutations in *POLR3A* and *POLR3B* are a major cause of hypomyelinating leukodystrophies with or without dental abnormalities and/or hypogonadotropic hypogonadism. J Med Genet.

[11] Dumay-Odelot H, Durrieu-Gaillard S, Da Silva D, Roeder RG, Teichmann M (2010). Cell growth- and differentiation-dependent regulation of RNA polymerase III transcription. Cell Cycle.

